# Exploring the Limitations of Hybrid Adiabatic Quantum Computing for Emission Tomography Reconstruction

**DOI:** 10.3390/jimaging9100221

**Published:** 2023-10-11

**Authors:** Merlin A. Nau, A. Hans Vija, Wesley Gohn, Maximilian P. Reymann, Andreas K. Maier

**Affiliations:** 1 Pattern Recognition Lab, Department of Computer Science, Friedrich-Alexander Universität Erlangen-Nürnberg (FAU), Martensstrasse 3, 91058 Erlangen, Germany; 2 Siemens Healthineers GmbH, Siemensstrasse 1, 91301 Forchheim, Germany; 3 Siemens Medical Solutions USA, Inc., 2501 Barrington Rd, Hoffman Estates, IL 60192, USA

**Keywords:** image reconstruction, quantum computing, quantum annealing, tomographic imaging, emission tomography

## Abstract

Our study explores the feasibility of quantum computing in emission tomography reconstruction, addressing a noisy ill-conditioned inverse problem. In current clinical practice, this is typically solved by iterative methods minimizing a $L^{2}$ norm. After reviewing quantum computing principles, we propose the use of a commercially available quantum annealer and employ corresponding hybrid solvers, which combine quantum and classical computing to handle more significant problems. We demonstrate how to frame image reconstruction as a combinatorial optimization problem suited for these quantum annealers and hybrid systems. Using a toy problem, we analyze reconstructions of binary and integer-valued images with respect to their image size and compare them to conventional methods. Additionally, we test our method’s performance under noise and data underdetermination. In summary, our method demonstrates competitive performance with traditional algorithms for binary images up to an image size of 32×32 on the toy problem, even under noisy and underdetermined conditions. However, scalability challenges emerge as image size and pixel bit range increase, restricting hybrid quantum computing as a practical tool for emission tomography reconstruction until significant advancements are made to address this issue.

## 1. Introduction

Quantum computing (QC) has become a popular topic in recent years due to its ability to handle complex computations faster than traditional methods [1]. Despite the limited practical applications of QC, it holds great promise for computation. The concept of “quantum supremacy” has been demonstrated twice in recent years, first in 2019 [2] and again in 2021 [3].

Quantum computers can be implemented with different physical principles despite utilizing the same underlying quantum mechanics. One widely recognized model is the gate-based model, as described in [1]. Other approaches include measurement-based, adiabatic quantum computing, as outlined in [4], and topological quantum computing.

We are making use of a D-Wave-produced adiabatic quantum computer, although it currently falls short of adhering to the adiabatic principles. As a result, the current implementation of the quantum computer is not universal. To overcome the limitations of the current quantum computer, we employ the use of hybrid solvers provided by D-Wave, which integrate both classical and quantum computing capabilities, as discussed in [5].

**Figure 1:**
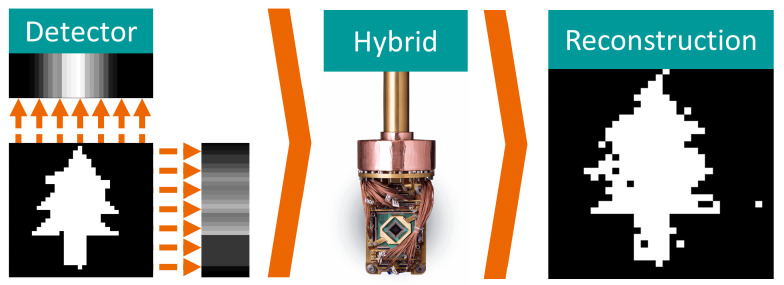
Graphical Abstract: Simulation and reconstruction of a two-view binary tomographic problem using hybrid quantum annealing.

In this study, we explore the application of quantum computing in tomographic image reconstruction; see the graphical abstract in Figure 1. Tomography, which refers to the imaging of cross-sectional views of an object, is a crucial tool in various fields, such as radiology, materials science, and astrophysics. In particular, we developed the method with an application in emission tomography (ET) [6] in mind. However, our tomographic model in this manuscript is in principle extendable to any tomographic imaging technique with a matrix-based system model. For simplicity, we may use the term tomography throughout this work.

The central challenge in tomographic imaging is to reconstruct the inner sections of an object. This constitutes an inverse problem, which is ill-posed. More to the point, loss of information or an increase in noise makes the problem hard to solve, as outlined in [6]. In the past, significant advancements in classical computing hardware were required to achieve the current level of image resolution of 512×512 in ET. With the continued development of quantum computing hardware, we anticipate that it will play a significant role in enhancing the performance of tomographic image reconstruction.

In the subsequent sections, we will provide an overview of quantum computing, with a specific focus on the D-Wave quantum computer used in our study. We will also delve into the basics of ET imaging and explain the inverse problem in tomographic reconstruction and current state-of-the-art methods for solving it. Then, we will elaborate on our approach to discrete tomographic reconstruction using hybrid quantum annealing (QA). Finally, we present results from our experiments, including small-scale reconstructed images with binary and integer values, test them in a variety of settings, and discuss the results.

## 2. Quantum Computing

QC is a new and innovative computing paradigm. Instead of using classical electronic bits, quantum computers utilize quantum bits (qubits) to exploit the quantum mechanical principles of superposition, entanglement, and interference. Using these paradigms, a quantum computer with *N* qubits can be in $2^{N}$ states simultaneously, compared to one state for a classical computer [7]. This advantage ultimately leads to a benefit in terms of runtime speed-up while enabling computations that are impossible on a classical computer. The concept of universal QC is accomplishable by several models. The total number of qubits is currently limited to 5640 qubits on the QA-based D-Wave Advantage2 system. For gate-based systems, the current record is set by the IBM Eagle system with 127 superconducting qubits [8].

### 2.1. Gate-Based Quantum Computing

Gate-based quantum computing utilizes a sequence or circuit of single-qubit and multiple-qubit gates to operate on the initial state of several qubits, similar to how classical gates operate on classical bits. Due to the exponential increase in Hilbert space, our Hilbert space doubles with every qubit that we add; we can achieve exponential speed-ups in particular algorithms [1].

### 2.2. Adiabatic Quantum Computing

The fundamental assumption of adiabatic quantum computing (AQC) is that a physical system constantly evolves to its lowest energy over time [2,9]—associated with the global minimum of an optimization landscape. In contrast to gate-based QC, AQC does not perform unitary operations through gates on single or multiple qubits [10]. Instead, we map the problem to the quantum computer with a problem-specific Hamiltonian [11]. The Hamiltonian describes the energy spectrum of the system and the set of viable solutions. In theory, AQC is equivalent to gate-based QC and, therefore, universal [4].

The adiabatic theorem states that if we initialize a quantum system with a ground state $\hat{H_{0}}$ and let it evolve with time *t* for a fixed duration *T*, we will end up in the ground state $\hat{H_{1}}$, which is associated with the lowest energy solution [12]:

(1) $$\hat{H}\left(t\right)=\left(1-\frac{t}{T}\right)\hat{H_{0}}+\frac{t}{T}\hat{H_{1}}.$$

The main limitation of AQC is the Δ gap. The Δ-gap refers to the minimum spectral gap of the problem’s Hamiltonian, which is the difference between the lowest and second-lowest energy levels. The Δ-gap inherently bounds the runtime of the adiabatic evolution. For further information, we guide the reader to [13]. The speed limit *T* is calculated as:

(2) $$T=\frac{1}{min{\left(\Delta \left(t\right)\right)}^{2}}.$$

### 2.3. Quantum Annealing

QA is the current realization of AQC. The QA system is initialized in a superposition state. Subsequently, the problem formulation is embedded in the hardware such that the system’s ground state is the solution to the problem [14]. However, how does QA overcome the Δ gap?

In short, it does not. Physical realizations of AQC usually let the system evolve multiple times for a specified time [15]. After initialization, the system repeatedly anneals for a specified annealing time $t_{a}$. This way, a sample set is formed containing the samples, the associated energy level (lowest is best), and the number of times the solution occurred [16].

QA builds upon the Hamiltonian of the Ising model:

(3) $$E_{Ising}\left(s\right)=\sum_{i=1}^{n}h_{i}s_{i}+\sum_{i=1}^{n}\sum_{i\neq j}^{n}J_{i,j}s_{i}s_{j}.$$

The variables $s_{i}$ of the Ising model, of *n* variables, are either spin-up or spin-down {−1,1} [1]. Two variables $s_{i}$ and $s_{j}$ can have quadratic interactions $J_{i,j}$, known as the coupling strength. Furthermore, one variable can have a linear bias $h_{i}$. Every Ising model is translatable to a Quadratic Unconstrained Binary Optimization (QUBO) problem, with variables $x_{i}$ being binary 0, 1, and vice-versa. QUBO problems can be NP-hard and are hard to solve using classical computers [17]. We can describe a QUBO using an n×n upper-triangular matrix with the bias term on its diagonal and the quadratic interaction as the upper-triangular values:

(4) $$f\left(x\right)=\sum_{i}Q_{i,j}x_{i}+\sum_{i<j}Q_{i,j}x_{i}x_{j}.$$

The goal is to minimize QUBO’s objective. In matrix notation, this results in the following:

(5) $$\min_{x\in {\left\{0,1\right\}}^{n}}x^{T}Qx.$$

**Figure 2:**
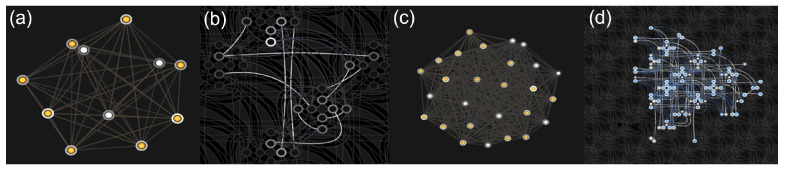
Graphs and physical embeddings on the QPU for binary reconstruction problems. (**a**,**c**) depict the directed graph for binary tomographic problems with image sizes of 4×4 and 8×8, respectively. (**b**,**d**) show the corresponding embedding of the directed graph on the QPU topology for (**a**,**c**), respectively.

The connectivity of the qubits on the annealer’s topology limits the interaction between qubits; see Figure 2. D-Wave has proposed different topologies over recent years, such as the Chimaera [18,19] or Pegasus graph. Using D-Wave’s Ocean interface [20], we can embed and run the problem on the quantum annealer using the Leap cloud service [21].

## 3. Emission Tomography

ET is a subset of tomographic imaging that encompasses two main modalities: Single Photon Emission Computed Tomography and Positron Emission Tomography [6]. In contrast to transmission tomography (TT), where the radiation source is external, and the radiation passes through the object, in ET, the object of interest emits radiation from within. In a medical scenario, this typically refers to the injection of a radioactive tracer into the patient’s body, which accumulates in a target destination and emits radiation.

The mathematical foundation of TT is rooted in the Radon transform, which is an integral transform that maps a continuous object to continuous measurements [22,23]. In ET, the basic Radon transform is extended to incorporate object-specific attenuation. The solution to the attenuated Radon transform is not straightforward but has been presented by Bronnikov and Natterer [24,25]. This, however, is outside of the scope of this paper.

The ET imaging process is similar to any linear digital imaging system, which maps a continuous domain to a discrete domain [26]. However, the image formation process is often simplified in a discrete-to-discrete forward model:

(6) y=Mx.

Here y represents our measurement, M is the system matrix describing the action of the linear imaging system, and x represents the imaged object. In practice, the system matrix M is sparse, exceptionally large, singular, ill-posed, and non-square and is therefore not usually applied as a simple matrix multiplication. It is important to mention that the system matrix M is only an estimate of the real forward operator, which is, in a real-case scenario, unknown. In reality, M is usually applied as a series of operators $M_{i}$, describing the relation from object space to measurement space.

(7) $$M=M_{k}⊗...⊗M_{1}.$$

The operators used in the imaging process allow for the inclusion of physical system-specific details, such as the point spread function, as well as operators to account for patient-induced attenuation and scatter. For this manuscript, we simplify the problem by disregarding the attenuation operator and use a deterministic system matrix model suitable for QA and their associated hybrid solvers. In the realistic case of an imperfect imaging system and setting, a common representation includes additive noise [26], although the noise can also be signal-dependent on x:

(8) y=Mx+n.

### 3.1. Discrete Tomography

Due to the nature of adiabatic quantum computing and its combinatorial optimization through QA, the obtained reconstruction data in quantum algorithms is inherently discrete. Unlike most classical algorithms that yield continuous results for the reconstructed image, quantum computing is currently limited in achieving floating-point accuracy, especially in its early stages of development. To bridge the gap between classical and quantum approaches, we propose to embrace the discretization of the reconstruction problem. By adapting tomography to discrete tomography, we take a bold yet essential step in aligning with the combinatorial nature of QA. In fact, discrete tomography is a specialized and simplified case of tomography, where the image x consists of discrete, or in the simplest case, binary pixels $x_{i}\in \left\{0,1\right\}$ [27] and has applications in real-world tomographic imaging. This case can apply to homogeneous scanning materials for non-destructive testing [27] or angiography in TT. Applications of DT in ET include attenuation-map reconstruction and cardiac and phantom imaging. We refer the reader to a manuscript by Herman et al. summarizing the application of DT in the medical domain [28]. Because the complexity of the optimization is constrained to discrete variables, the task’s difficulty is to reconstruct with as few views as possible. In our experimental setup, we focus on discrete tomography scenarios, as these problems are directly applicable to QA and associated hybrid solvers available at present.

### 3.2. Image Reconstruction

ET reconstruction is in the class of ill-conditioned noisy inverse problems and constitutes the problem of resolving an activity distribution that generated the measured projection data. Due to the reasons mentioned earlier, obtaining an exact inverse of M is not possible. Therefore, one conventional clinical method to reconstruct ET images is Filtered BackProjection (FBP), an analytical and linear approach [29]. More advanced approaches to reconstructing ET images are iterative reconstruction algorithms [30]. In contrast to FBP, these algorithms are often non-linear and seek to minimize the projection difference by repetitively applying back projections, updates, and forward projections [31]. Three examples of iterative reconstruction techniques include Maximum Likelihood Expectation Maximization [31,32], Conjugate Gradient [33], and Simultaneous Algebraic Reconstruction Technique (SART) [34]. SART is an algebraic iterative reconstruction algorithm that performs additive and iterative updates from single projections. A popular reconstruction algorithm for discrete tomography is the Discretized Algebraic Reconstruction Technique (DART), which is an extension of the SART algorithm. Recently, deep-learning-based image reconstruction [35] has gained popularity, and we acknowledge its success. However, the primary focus of this manuscript is not to contest deep-learning methods but rather to explore the application of image reconstruction on quantum computers. As the image size for the QA-based reconstruction is inherently limited by the size of the annealer, we also consider the Moore-Penrose general pseudoinverse (PI) as a reconstruction technique. The Moore-Penrose PI is defined by [36]:

(9) $$M^{†}={\left(M^{T}M\right)}^{-1}M^{T}.$$

For this work, we have chosen to compare our method to an FBP, DART, and a Moore-Penrose-based PI [36] image reconstruction. Due to the fact that both FBP and PI result in a continuous image, we observe a numerical error when compared to discrete reconstruction techniques, which we mitigate by introducing a subsequent discretization step. We compare the reconstructed images and their corresponding ground truth in terms of the root mean square error (RMSE), which measures the average magnitude of the differences between reconstruction and ground truth:

(10) $$\mathrm{RMSE}\left(\hat{x},x\right)=\sqrt{\frac{1}{n}\sum_{i=1}^{n}{\left({\hat{x}}_{i}-x_{i}\right)}^{2}}.$$

Here $\hat{x}$ represents the reconstructed image, whereas x represents the corresponding ground truth. Moreover, we compare the reconstructed images in terms of their Structural Similarity Index Measure (SSIM), which measures the quality of a reconstructed image in terms of their luminance, contrast, and structure as a mean over a moving window [37]:

(11) $$\mathrm{SSIM}\left(\hat{x},x\right)=\frac{\left(2\mu_{\hat{x}}\mu_{x}+c_{1}\right)\left(2\sigma_{\hat{x}x}+c_{2}\right)}{\left(\mu_{\hat{x}}^{2}+\mu_{x}^{2}+c_{1}\right)\left(\sigma_{\hat{x}}^{2}+\sigma_{x}^{2}+c_{2}\right)}$$

For the computation of the SSIM, $\mu_{\hat{x}}$ and $\mu_{x}$ are the average pixel values of images $\hat{x}$ and x, respectively. $\sigma_{\hat{x}x}$ is the covariance between images $\hat{x}$ and x and $\sigma_{\hat{x}}^{2}$ and $\sigma_{x}^{2}$ are the variances of images $\hat{x}$ and x, respectively. $c_{1}$ and $c_{2}$ are small constants to stabilize the division. The SSIM represents a unitless index measure indicating if images are similar (SSIM of 1), dissimilar (SSIM of 0), or anti-correlated (SSIM of −1).

## 4. Related Work

Over recent years, QC research has accelerated rapidly as practical examples have come within reach. Specifically, image processing and machine learning on quantum computers have evolved to be an active area of research [38,39]. Here, relevant work of quantum algorithm development for tomographic image reconstruction should be highlighted.

The first who proposed to perform image reconstruction with QC for TT, positron ET, and magnetic resonance imaging were Kiani et al. [40]. The paper proposed to substitute the classical Fourier transform with a quantum-based Fourier transform (QFT) for inverse integral transform-based reconstruction methods to decrease runtime [40]. However, integral transform-based reconstruction, in contrast to analytic algorithms, fail to incorporate additional knowledge of the physical measurement process and the imaging system itself. Therefore, the incorporation of a system-specific matrix or operator is crucial to the success of an image reconstruction algorithm, which can then be posed as the solution to a linear system of equations. The initial proposal to solve linear equations on a gate-based quantum computer was made by Harrow et al. in 2009 when they introduced the Harrow-Hassidim-Lloyd (HHL) algorithm [41]. The HHL algorithm is based on quantum phase estimation and, in theory, provides logarithmic speed-up against classical computers. Nonetheless, there are a few caveats for which situations this speed-up is lost [42]. The first caveat is the loading of the vector y into a quantum state. This also applies to the QFT approach. The second caveat is that the matrix M needs to be sparse, and the third is that the matrix M needs to be robustly invertible, which is associated with the ratio of the largest and smallest eigenvalues and therefore limits the application to low-conditioned matrices. The last caveat is associated with the readout of the solution vector x. This would, in practice, have to be measured *n* times, again ruining the proposed speed-up. Therefore, the HHL algorithm is not efficient to run on near-term quantum hardware, also due to the current qubit count and error. We, therefore, disregard this approach.

Another take to solving (combinatorial) optimization problems on near-term gate-based quantum computers is the Quantum Approximate Optimization Algorithm (QAOA) [43], which is similar to QA-based systems in its problem formulation. The first example presented for QA was non-negative binary matrix factorization [44]. Furthermore, Chang et al. demonstrated in 2019 that it is possible to solve polynomial equations with QA [45]. The approach was refined to a linear system with floating-point values and floating-point division by Roger and Singleton in 2020 [46]. Their paper utilizes the D-Wave 2000Q system to its whole extent and shows results for matrix inversions of 3×3 matrices. However, they fail for matrices with high condition numbers. The first practical application to utilize QA for linear systems was Souza et al., who presented a seismic inversion problem, which they solved in a least-square manner [47]. Schielein et al. presented a road map towards QC-assisted TT, describing data loading, storing, image processing, and image reconstruction problems [48]. For a long time, QA hardware needed to be more mature for realistic problems. Schielein et al. and Jun also proposed to solve tomographic reconstruction with QA or QAOA [48,49].

In this work, we want to present multiple achievements:

- Comparison of binary and integer-based tomographic reconstruction run on actual QC hardware to classical reconstruction algorithms on small-scale images in a toy model
- Analysis of capabilities and limitations of QA regarding image size, discretization, noise, underdetermination, and inverse crime of the reconstruction algorithm
- A framework for the creation of tomographic toy problems to accelerate quantum image reconstruction research

## 5. Methods

This section will cover how we solve an inverse problem with QA. We cover the fundamentals of embedding the problem on the quantum processing unit’s (QPU) topology. We elaborate on the limitations of the current topology and how hardware must advance to run the optimization of problems at a significant scale. Furthermore, we describe how hybrid algorithms can utilize QA to its full extent and how we must embed the problem for the hybrid approach.

### 5.1. Problem Formulation

We recall that the reconstruction problem in tomography is an inverse problem of the form shown in Equation (6). Because our system of equations is an ill-posed image reconstruction problem that contains noise and may not be fully determined, we approximate the solution in a least-squares manner. Therefore, we reformulate Equation (6) as the objective of a quadratic minimization problem, with its minimum being the approximated solution of image x:

(12) $$\begin{array}{c} H\left(x\right)={∥Mx-y∥}_{2}^{2}=x^{T}M^{T}Mx-x^{T}M^{T}y-y^{T}Mx+y^{T}y. \end{array}$$

### 5.2. Quadratic Unconstrained Binary Optimization for Binary Tomography

In the QC fundamentals, we have discussed that the Ising model is the basic Hamiltonian for QA. In the case of binary tomography, the reconstruction problem is directly mappable to a QUBO problem. We recall that the binary tomographic model is defined like Equation (6), where $M\in R^{\left(m\times n\right)}$, $y\in R^{\left(m\right)}$ and $x\in {\left\{0,1\right\}}^{n}$. The linear bias values can be extracted by following Equation (12):

(13) $$Q_{i,i}=-2\sum_{j}y_{j}M_{ji}.$$

Furthermore, we can extract the coupler values as:

(14) $$Q_{i,j}=\sum_{k}M_{ki}M_{kj}.$$

The offset ${∥y∥}^{2}$ does not change the minimization problem’s objective. The overview of the reconstruction process is as follows: We initialize the quantum annealer with the obtained linear biases and quadratic couplers, specify the number of times the system anneals, and then obtain a sample set containing the number of solutions, each associated with an energy. The solution to the minimization problem is then chosen as the one corresponding to the lowest energy. The optimization scheme is described in Algorithm 1.
**Algorithm** **1:** Quantum annealing tomographic reconstructionData: $M\in R^{m\times n}$: System matrix   $y\in R^{m}$: Projection vectorParameter: number_reads: Number of annealing repetitionsResult: $x\in {\left\{0,1\right\}}^{n}$: Image vector$\mathrm{Linear}\_\mathrm{Coefficients}\leftarrow -2y^{T}M$$\mathrm{Quadratic}\_\mathrm{Coefficients}\leftarrow M^{T}M$offset $\leftarrow y^{T}y$bqm ← CreateBQM(Linear_Coefficients, Quadratic_Coefficients, offset)// Scale chain strength of quantum annealer depending on problemchain_strength ← scaleChainStrengthToBQM(bqm)// Run BQM on quantum annealersampleset ← SampleBQMOnQuantumAnnealer(bqm, number_reads, chain_strength)// Retrieve sample associated with the lowest energyx← sampleset[0]return x

When directly mapping a QUBO problem to the quantum annealer, one must consider the embedding of the QPU’s topology. The qubits on a Chimaera topology, as embedded on the D-Wave 2000Q, are internally connected to four other qubits and have one or two external connections to other qubits. The newer D-Wave Advantage2 system features internal connectivity of one qubit to 12 other qubits and two to three external couplers. To map higher connectivity graphs to the QPU, one utilizes chains of physical qubits to represent one logical qubit. Fully connected graphs, like our problem, are mapped to the QPU using a clique embedding [18]. The most extensive, mappable, fully connected graph on the QPU is a graph of 65 logical qubits on the D-Wave 2000Q and 100 logical qubits on the D-Wave Advantage2. Our binary reconstruction problem, now defined as the QUBO matrix Q, is fully connected. More to the point, variables usually have quadratic interactions with all other variables. The limit in terms of reconstructed binary image size for the D-Wave Advantage2 is 10×10. Therefore, using a QA to reconstruct a *R*-bit integer image of size N×N without problem optimization will require $N^{2}R$ fully connected qubits. This qubit amount and connectivity will not be available soon. Figure 2 shows examples of the graph and corresponding embedding for image sizes of 4×4 and 8×8. In Figure 2, the directed graph represents the structure of the QUBO problem, where one vertex is a binary variable, or logical qubit, with a corresponding linear bias. The edges represent the quadratic couplings between the variables. The physical embedding shows how the directed graph is mapped to the QPU topology using a clique embedding. Here, the marked vertices symbolize the physical qubits that are used to embed the directed graph. The edges, again, represent the quadratic couplings. For a more detailed description of how problem graphs are embedded in the corresponding hardware, we refer the reader to [50]. To embed more significant problems on the D-Wave machine, we use hybrid algorithms, which are defined below.

### 5.3. Hybrid Optimization

The intermediate step to complete quantum-assisted computation is to design hybrid algorithms to enable the embedding of significant large problems on current QC hardware. On quantum annealers, one can make use of hybrid workflows. Raymond et al. [5] introduced one type of hybrid computation. Their algorithm uses a large neighborhood local search to find subproblems in the original problem. The subproblems are then of a size that is mappable to the QPU. To enable the solution of larger quadratic problems that cannot be run efficiently on the quantum annealer, D-Wave has introduced a commercial hybrid solver available through the Leap cloud service [21]. The D-Wave hybrid solver represents a devised solution that combines their primary quantum annealing approach with advanced classical algorithms. This hybrid solver efficiently allocates the quantum processing unit (QPU) to where it offers the most advantages in problem-solving. Moreover, one can utilize the hybrid solver to solve constrained quadratic models (CQM), which enable the use of integer values and, therefore, drastically expand solution possibilities. The new constrained quadratic model is defined as:

(15) $$H\left(x\right)=\sum_{i=1}^{n}a_{i}x_{i}+\sum_{i=1}^{n}\sum_{i\neq j}^{n}b_{ij}x_{i}x_{j}+c.$$

Here, $x_{i}$ is the unknown integer variable (pixel value) we want to optimize for, $a_{i}$ is the linear weight, $b_{i,j}$ is the quadratic term between $x_{i}$ and $x_{j}$ and *c* can define possible inequality and equality constraints. In principle, the workflow is defined by the classical problem formulation and a time limit *T* [51]. The time limit *T* is automatically calculated depending on the problem if not specified by the user. The solvers run in parallel and utilize heuristic solvers to explore the solution space and then pass this information to a quantum module that utilizes D-Wave systems to find solutions. The QPU solutions then guide the heuristic solvers to find better quality solutions and restrict the search space. This process iteratively repeats for the specified time limit. Furthermore, one has the possibility of introducing quadratic and linear constraints and restricting the range of the integers [51]. Similar to QA, one obtains a set of solutions, each associated with energy, where the solution associated with the lowest energy is the best-approximated solution to the minimization problem. The optimization scheme is described in Algorithm 2.   
**Algorithm** **2:** Hybrid quantum annealing tomographic reconstructionData: $M\in R^{m\times n}$: System matrix   $y\in R^{m}$: Projection vectorParameter: time_limit: Time limit for the optimizationResult: $x\in Z_{\geq 0}^{n}$: Image vectorx← [$pixel\_integer_{0},...,pixel\_integer_{n}$]objective $\leftarrow {\left(\mathrm{Mx}-y\right)}^{2}$cqm ← CreateCQM(objective)cqm.add_constraint(x≥0)// Run CQM on hybrid solversampleset ← SampleCQMOnHybridSampler(cqm, time_limit)// Retrieve sample associated with lowest energyx← sampleset[0]return x

## 6. Results

In this section, we present novel reconstruction results of our algorithm utilizing D-Wave’s quantum computers and compare them to classical methods. Due to the size restrictions on the actual quantum annealer, images are reconstructed using hybrid solvers to enable the representation of more significant problems. Every hybrid reconstruction is compared to three different classical methods: discretized FBP, DART, and discretized PI. We discretize the reconstruction result of the classical algorithms to compare them in the case of binary and discrete tomography. We refer to our hybrid reconstruction method as QA. Moreover, we compare the ground truth image (GT) to the reconstructions and visualize the corresponding sinogram (SG) to the tomographic problem. We utilize SymPy for our problem formulation and solve the reconstruction problem as a classical forward and inverse problem Mx=y. Therefore, it, in principle, applies to any linear imaging or display system. The system matrix for the reconstruction problem is calculated using the Radon transform. We acknowledge that for more realistic and significant problems, the system matrix becomes infeasible to store, and our approach is not directly applicable. Nevertheless, we want to test the general performance of hybrid solvers utilizing QA on inverse problems regarding size, noise, and underdetermination of the linear equations, even at the very beginning of practical QC.

### 6.1. Experimental Setup

We have constructed a tomographic toy problem framework to test quantum computers’ initial stages of image reconstruction. We set up example problems for our QA-based reconstruction by generating tomographic problems in a linear system manner. We utilize scikit-image [52] to perform Radon transforms of our GT images and create our system matrices. Here, the integration of the object rotated by an angle α defines one projection view. The number of angles is equally distributed between $0^{\circ }$ and $180^{\circ }$. ET inspires our model. In contrast to TT, we do not model the attenuation of a source light ray through the object. Instead, we aim to model the emission process of photons (counts) within the object. For now, we are neglecting the attenuation of photons by the object. This aspect may be addressed in future research. The projection views at $0^{\circ }$ are taken from the top of the image. Subsequently, the angles are distributed in a clockwise direction. We have utilized scikit-image’s iradon to perform FBP with a ramp filter and modified sart to a DART reconstruction algorithm. For DART, we perform two iterations of the algorithm. The PI-based reconstruction uses NumPy’s pinv function to estimate a Moore-Penrose PI. We utilize scikit-image’s structural_similarity function to quantify SSIM, using a default window size of 7×7 for comparison and adapting it to 3×3 for the smallest image size. Furthermore, we introduce an uncertainty map (UC) for our proposed reconstruction technique. The UC displays the pixel-wise variance of all returned samples by the hybrid sampler, independent of their associated energy. This UC will only be highlighted in the cases where the uncertainty is reasonably high.

#### Additive Noise

To test problems concerning noise in the data, we establish a simple noise model to alter the projection data. Now, we want to imitate the statistics of a low-count ET measurement. We apply the noise in an additive manner to the GT image for each projection view to create independent noise realizations:

(16) $$x_{noise}=x+n.$$

The noise is therefore defined as follows:

(17) $$n_{i}=\left\{\begin{array}{c} -1,0,1,x_{i}\neq 0\\ 0,1,\mathrm{if}x_{i}=0 \end{array}\right..$$

With this noise model, we want to resemble the signal dependence of Poisson noise. Poisson noise is the most prominent noise factor in projection images with very low counts.

### 6.2. Experiment 1: Image Size Evaluation

**Figure 3:**
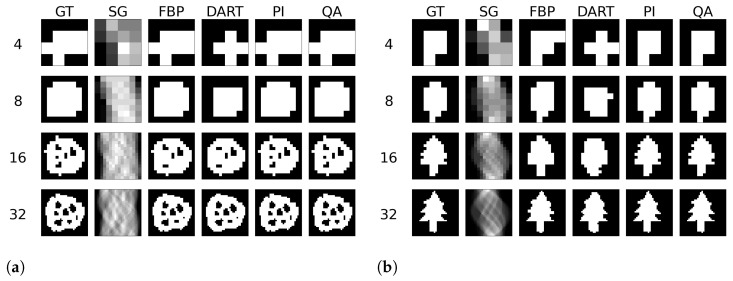
Binary reconstructions of sample image ‘foam’ (**a**) and ‘tree’ (**b**) for image sizes N×N, where *N* is 4, 8, 16, and 32.

**Figure 4:**
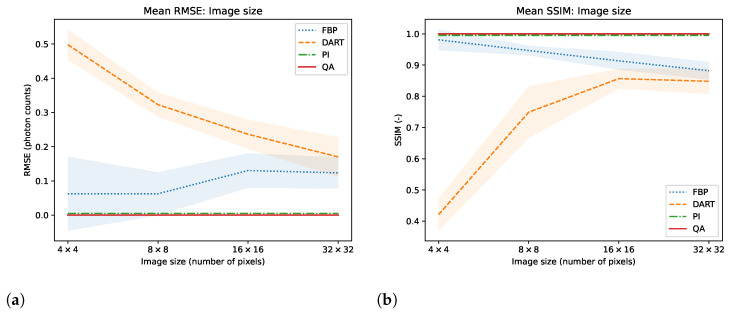
Mean and variance RMSE (**a**) and SSIM (**b**) evaluation of images ‘foam’, ‘molecule’, ‘snowflake’ and ‘tree’ for image sizes N×N, where *N* is 4, 8, 16, and 32.

We test the reconstruction capabilities of the hybrid solver concerning the image size N×N of x. Algorithm 2 describes the problem formulation for the hybrid solver. We apply our reconstruction technique to four different binary images ‘foam’, ‘tree’, ‘snowflake’, ‘molecule’ at four different squared image sizes 4, 8, 16, and 32. We chose the images to achieve a variance in frequency and image content. To downsample the image, we take local means of image blocks. We take *N* projection view with *N* measurements for each view. Thus, we have a fully determined system for image size N×N. We neglect the problem of ‘inverse crime’ for this experiment and test the algorithm in its simplest form. We show the reconstructed images and their corresponding GT, SG, and comparable classical results for the binary images ‘foam’ and ‘tree’ in Figure 3. The examples for ‘snowflake’ and ‘molecule’ are in the Appendix A.1. Moreover, we compare the reconstruction algorithms on the four binary images measured by root mean square error (RMSE) and structural similarity index (SSIM) in Figure 4. A comparison of the reconstruction runtimes is provided in Table 1.

**Figure 5:**
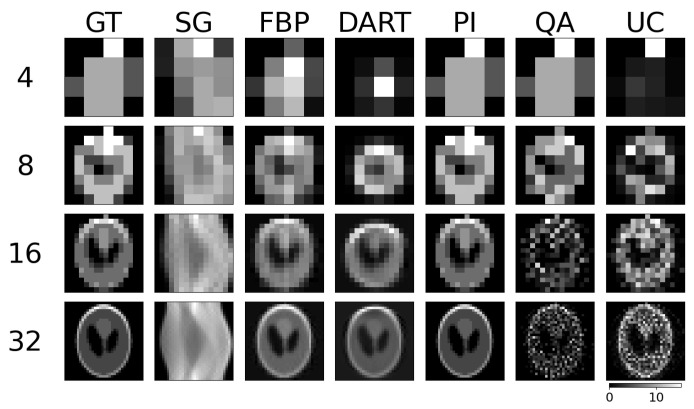
4-bit integer reconstructions of Shepp–Logan phantom for image sizes N×N, where *N* is 4, 8, 16, and 32.

**Figure 6:**
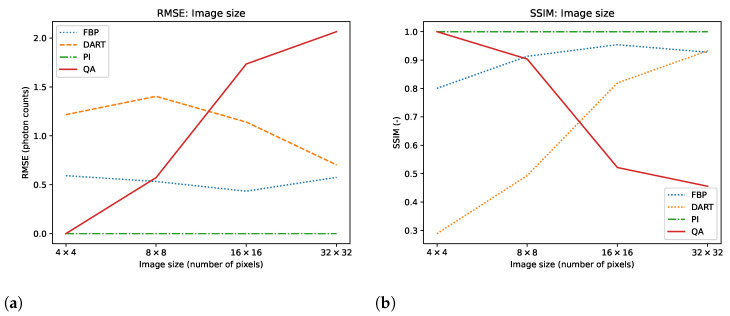
RMSE (**a**) and SSIM (**b**) of 4-bit integer reconstructions of the Shepp–Logan phantom for image sizes N×N, where *N* is 4, 8, 16, and 32.

As a subsequent experiment, we employ the hybrid solver to solve for integer-valued variables in a 4-bit range representing the numbers from 0 to 16. With this, we move towards a more realistic use case. We simulate the well-known Shepp–Logan phantom at the possible 4-bit range, compare the hybrid integer reconstructions with conventional reconstruction methods, and visualize the UC in Figure 5. Furthermore, we plot a comparison regarding RMSE and SSIM for the reconstructed image size in Figure 6. Additionally, we provide a more thorough component-wise analysis of the Shepp–Logan phantom in the Appendix A.2, which increases the discretization values step-by-step.

### 6.3. Experiment 2: Noise Evaluation

**Figure 7:**
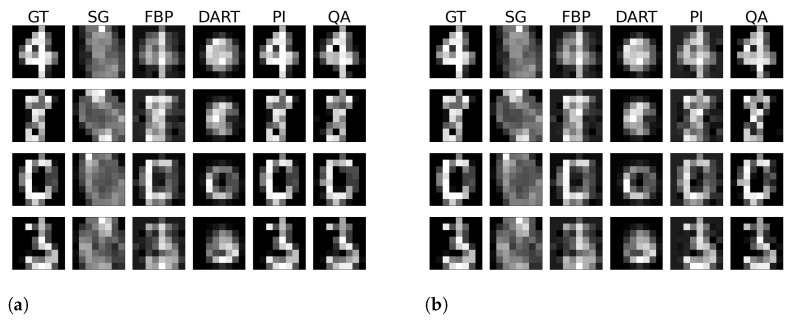
4-bit integer reconstructions of four digits from the UCI digits dataset without (**a**) and with random noise (**b**).

**Figure 8:**
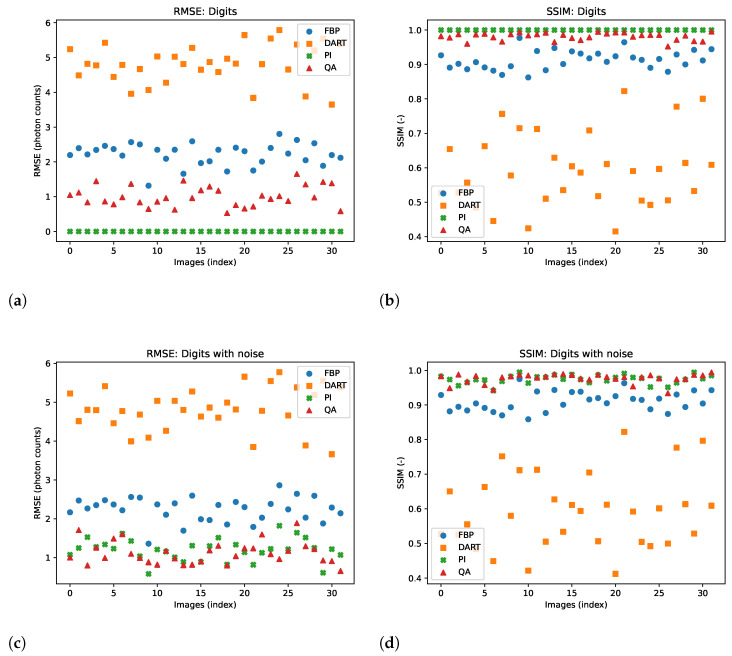
Comparison of RMSE and SSIM for 4-bit integer reconstructions of 32 images from the UCI digits dataset. (**a**,**b**) show results without noise, while (**c**,**d**) show results with noise.

One typical problem in image reconstruction is the noise in the measured data. Especially in low-count tomography, one suffers from high photon noise. We alter the image data with our noise model to imitate the high-noise level in low-count ET, which addresses the problem of ‘inverse crime’. In comparison to the previous experiments, we use a truncated PI with a cut-off value of 0.001 to make the corresponding reconstruction robust to the noise induced. We test the hybrid-based reconstruction’s robustness with a simple noise alteration of the GT image during the acquisition. We utilize the UCI Machine Learning Repository Digits dataset [53] for small-scale images with low bit range, as we see the limitations of the approach in the Shepp–Logan phantom. The dataset consists of 5620 digits of image size 8×8 with a bit range of [0, 16]. We randomly chose 32 digits and reconstructed them with and without noise. The additive noise is described in Equation (16). Visual results of the reconstructed images without and with noise are shown in Figure 7. Again, we show the reconstructed images and their corresponding GT, SG, and comparable classical results. The remaining reconstructed digits can be found in the Appendix A.3 and Appendix A.4. Furthermore, we present a quantitative evaluation of the RMSE and SSIM for both noise-free and noisy data for each digit image in Figure 8.

### 6.4. Experiment 3: Underdetermined Evaluation

**Figure 9:**
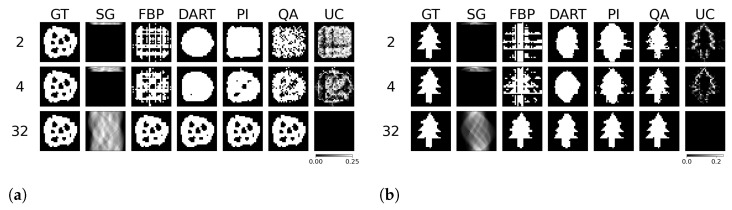
Binary reconstruction of the 32×32 image ‘foam’ (**a**) and ‘tree’ (**b**) from 2, 4, and 32 views.

**Figure 10:**
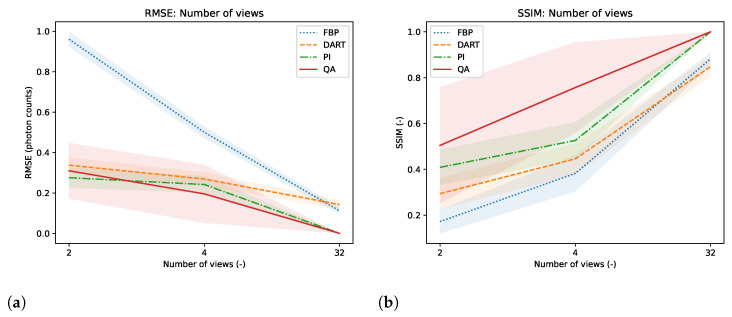
RMSE (**a**) and SSIM (**b**) of binary reconstructions from 2, 4, and 32 views.

The reconstruction of binary images in a fully determinant setting is easy for any reconstruction algorithm, as the number of combinatorial options is minimal compared to integer or floating-point-based reconstruction. The problem in binary tomography primarily results from reconstructing the objects with as few views as possible. In the past, methods have been presented to reconstruct an object with two views only [27]. The methods usually enforce much regularization and prior knowledge of the object due to the associated null space of the projection operator in few-view reconstruction. Therefore, we want to present reconstruction results of binary images of size 32×32 with only 4 and 2 projection views acquired while only enforcing the prior knowledge of assuming binary pixels. The reconstructed images, their comparison algorithm results, and corresponding GT, SG, and UC are displayed for the binary image ’foam’ and ’tree’ in Figure 9. The other examples are provided in the Appendix A.5. Moreover, we compare the reconstruction algorithms on the four binary images measured by RMSE and SSIM in Figure 10.

### 6.5. Experiment 4: Inverse Crime and Applications towards Reality

**Figure 11:**
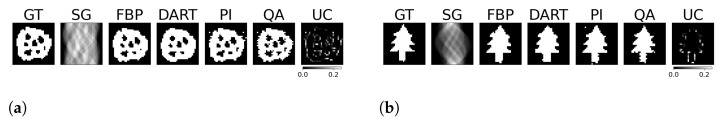
Outside of the inverse-crime scenario: Binary reconstructions of sample image ‘foam’ (**a**) and ‘tree’ (**b**) for image size 32×32. The projections are simulated on an upscaled higher-resolution image 128×128 of the input, and the sinogram is consequently rebinned.

In our previous experiments, we assumed the exact system matrix for precise forward projection. This allows us to evaluate the algorithm in a controlled environment with simulated perfection. However, real-world situations rarely provide a perfect system matrix, and we often rely on estimated versions instead. To prevent biases caused by the ’inverse-crime’ scenario, we now simulate the images on a different, higher-resolution grid during simulation. To simulate these scenarios, we take the previously presented binary images and scale them up to a size of 128×128. We then simulate the projections and perform the reconstructions using a rebinned SG. Again, we compare against a truncated PI in this case. The reconstructed images, their comparison algorithm results, and corresponding GT, SG, and UC are displayed for the binary image ’foam’ and ’tree’ in Figure 11. The remaining examples are provided in the Appendix A.6. A comparison of the mean RMSE and SSIM is given in Table 2.

## 7. Discussion

Compared to previous approaches for matrix inversion based on QA, we observe an improvement in linear systems with high condition numbers as well as the size of the linear system itself. Rogers and Singleton’s method of solving linear systems using a quantum annealer is restricted to small systems, 3×3, with very low condition numbers [46]. With the use of hybrid solvers, we can overcome this issue. Our system matrices M are singular, with a condition number approaching infinity. When entirely determined, the results of small binary tomographic reconstruction contest against conventional methods. We hypothesize that the hybrid solver can handle binary image sizes of up to 32×32 without difficulty; see Figure 3. With integer-based tomographic reconstructions, we encounter challenges with larger image sizes exceeding 8×8, as observed from Figure 5. For this reason, we further performed integer-valued tomography reconstruction only for the digits dataset with image size 8×8 in Figure 7. Here, we can see that hybrid-based reconstruction can yield similar results to the conventional algorithms. An exciting finding is evident when comparing the RMSE and SSIM of noisy simulations. The hybrid-based reconstructions are robust to noise and yield similar performance to the conventional reconstruction technique for all 32 digits in this small-scale experiment, as displayed in Figure 8. We acknowledge that the energy optimization landscape appears to be minimally affected by noise during the hybrid annealing process. However, further research is warranted to gain a deeper understanding of this phenomenon. Moreover, we see that the reconstruction from as few views as 2 or 4 projections can outperform standard reconstruction algorithms in binary-based images in this specific toy example. However, the variance of the quantitative results is relatively high, as seen in Figure 10. The high variance can be attributed to the reconstructed images, which have higher frequency content, particularly within the imaged object. Finally, we observe comparable results to conventional algorithms in a more realistic binary reconstruction scenario, where the system matrix is not precisely known.

At this point, it is important to mention that the conventional reconstruction techniques are not designed for such small-scale images, and the comparison may not accurately reflect how the algorithm will compare on larger image sizes, especially in the case of real ET reconstruction with non-discrete value. This is particularly manifested in the observation that the error of conventional reconstruction methods minimizes when approaching larger image sizes, as seen in Figure 4 and Figure 6. This can be attributed to the fact that both FFT and DART utilize linear interpolation in their reconstruction process. In contrast, both QA and PI employ the direct system matrix, which mitigates these errors. However, for the current state-of-the-art QC, it is infeasible to conduct such experiments, and we still see great value in the experiments with such new computing technologies. If quantum annealing can be scaled up to reasonable image sizes and pixel value ranges in the future, it may offer advantages. These advantages include increased robustness to noise and the ability to reconstruct with fewer views. In a realistic scenario, this could translate into reduced radiation exposure for patients and shorter scanning times, but this remains an open research question for evaluation in the future. We also see potential drawbacks of our method. Most importantly, the D-Wave quantum annealer and the associated hybrid solvers are no universal quantum computers. Therefore, we can only perform the QA algorithm on the hardware. In return, this means that we cannot use the ability of quantum computers to represent extensive data with significantly fewer qubits. On the other hand, the data loading is part of the problem formulation for QA, which is a time-consuming task for gate-based QC. However, there is no guaranteed speed-up or better solutions proofed for QA [54]. The use of hybrid solvers helps to improve the quality of solutions and problem size. However, the problems may require a long runtime, which cannot compete with purely classical methods. In the hybrid approach, a majority of the runtime is attributable to the overhead of classical computation. Another drawback is the current cost of QC, which is relatively high but is expected to decrease as it did for classical computers.

Finally, we observe that the hybrid-based integer reconstructions have problems reconstructing homogeneous regions. Adding smoothness constraints to the objective could improve reconstructed images in the future.

## 8. Conclusions and Outlook

We have given the reader an overview of quantum computing, especially adiabatic quantum computing. More to the point, we have explained how quantum annealing works and which problems it can solve. Subsequently, we present the inverse problem of tomographic image reconstruction and describe the use case of emission tomography and the difference to transmission tomography. We summarize previous work in the solution of linear systems and image reconstruction with quantum annealing and quantum computing and provide the fundamentals for our reconstruction method with quantum annealing and hybrid solvers. Finally, we showcase the results of binary- and integer-valued reconstruction for different matrix sizes. We also test the reconstruction concerning noise and underdetermination. We have observed that hybrid-based reconstruction shows potential benefits in noisy linear systems, especially for binary images and very small integer-valued images, where the problem’s solution space is still tractable for the hybrid solver. However, challenges arise as we increase the pixel bit range of images, and we do not observe performance comparable to that of classical techniques. Furthermore, the preparation of larger optimization problems, which is carried out on a classical computer, becomes a bottleneck in the reconstruction pipeline due to the increasing size of the system matrix used for reconstruction. Nevertheless, the stochastic nature of quantum annealers allows for the introduction of additional uncertainty measures to interpret the reconstructed images. 

## Figures and Tables

**Table 1:** Overview of the reconstruction times for the hybrid QA-based reconstruction technique compared to classical reconstruction methods FBP, DART, and PI. FBP, DART, and PI were carried out on a developer’s workstation with an Intel^®^ Core™ i7-10850H CPU. The runtime of the hybrid QA-based reconstruction is subdivided into the creation of the binary quadratic model (BQM) on both the developer workstation and the commercial hybrid solver and then the subsequent CPU and QPU optimization carried out on the commercial hybrid solver. The time is measured as the mean of four 32×32 binary reconstructed images.

Reconstruction	Create BQM (s)	CPU Opt. (s)	QPU Opt. (s)	Total Runtime (s)
FBP	-	0.002	-	0.002
DART	-	0.007	-	0.007
PI	-	0.641	-	0.641
QA	38.22	5.095	0.032	43.347

**Table 2:** Overview of the RMSE and SSIM measured as the mean of the four 32×32 binary reconstructed images outside the inverse-crime scenario.

	FBP	DART	PI	QA
RMSE (mean + std)	0.281±0.035	0.279±0.038	0.289±0.044	0.279±0.039
SSIM (mean + std)	0.629±0.050	0.623±0.050	0.609±0.066	0.638±0.035

**Figure A1:**
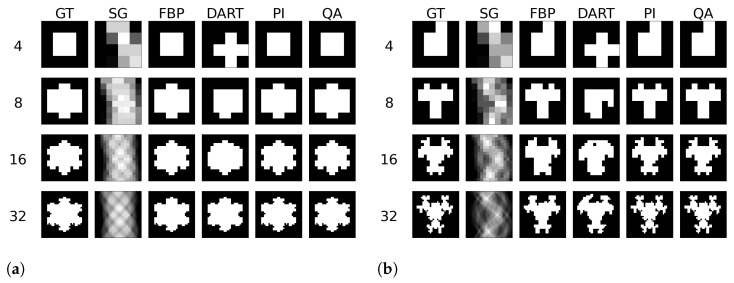
Binary reconstructions of sample image ‘snowflake’ (**a**) and ’molecule’ (**b**) for image sizes N×N, where *N* is 4, 8, 16, and 32.

**Figure A2:**
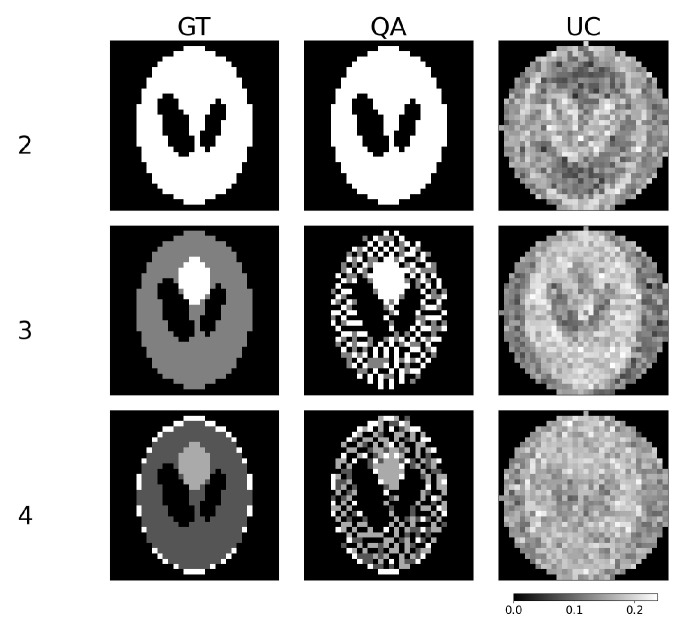
Gradual analysis of the Shepp–Logan phantom. The y-axis shows the number of components (discretization values) in the Shepp–Logan phantom.

**Figure A3:**
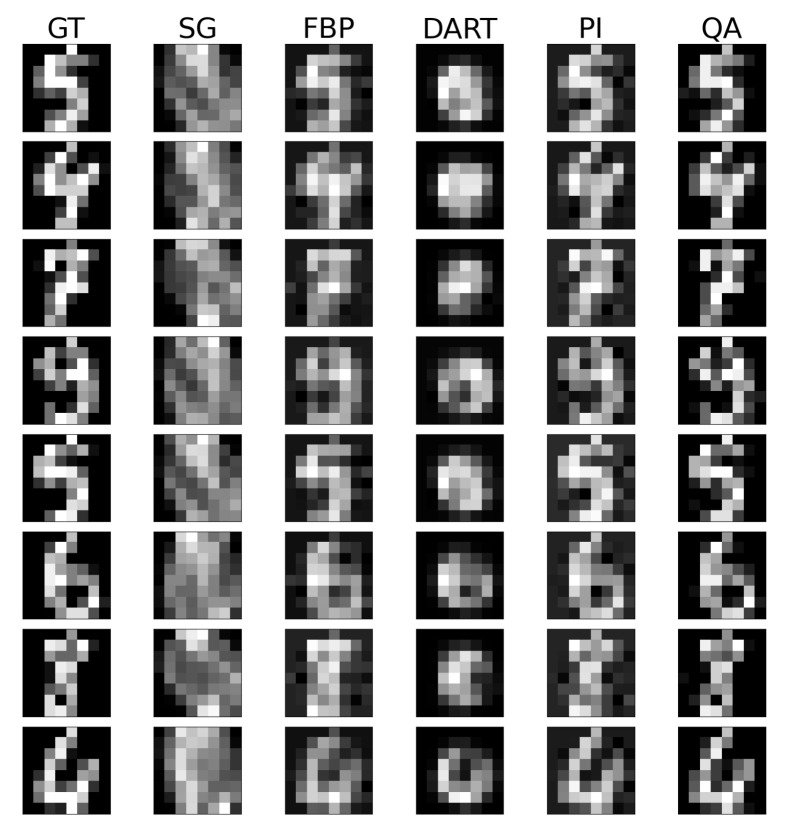
4-bit integer reconstructions of digits from the UCI digits dataset.

**Figure A4:**
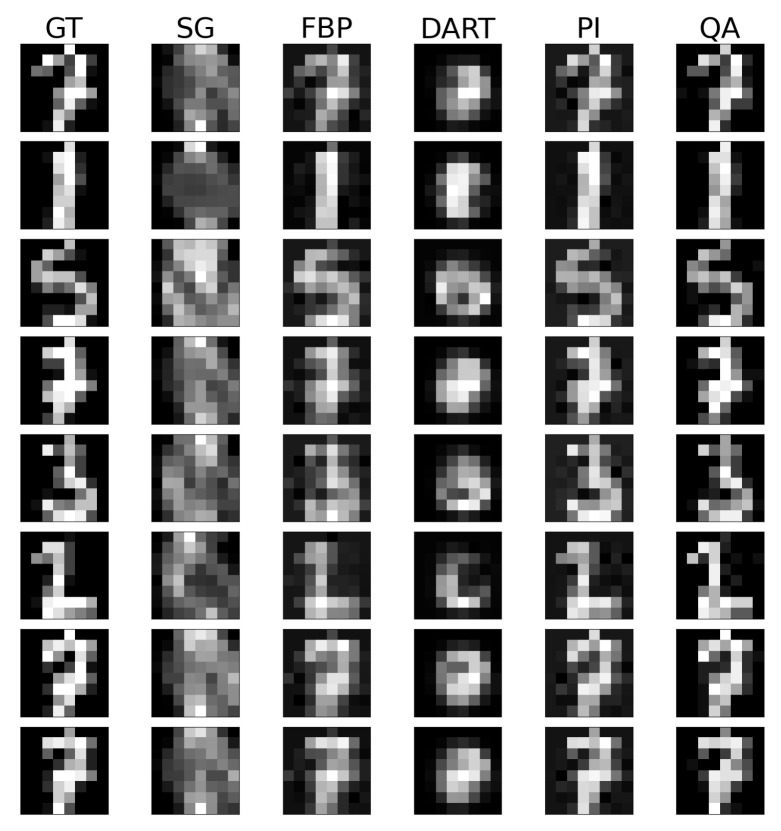
4-bit integer reconstructions of digits from the UCI digits dataset.

**Figure A5:**
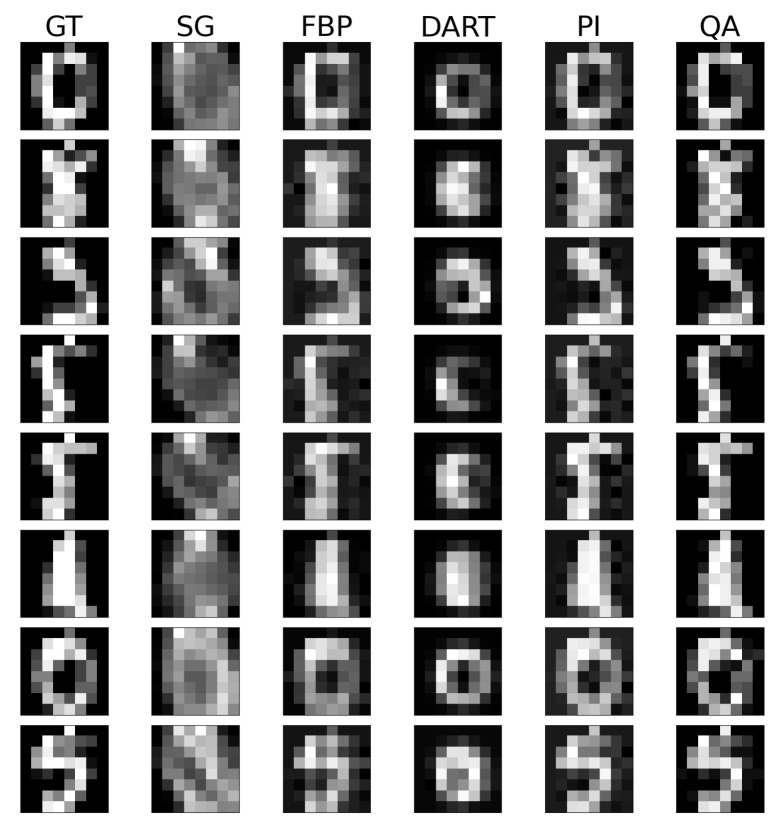
4-bit integer reconstructions of digits from the UCI digits dataset.

**Figure A6:**
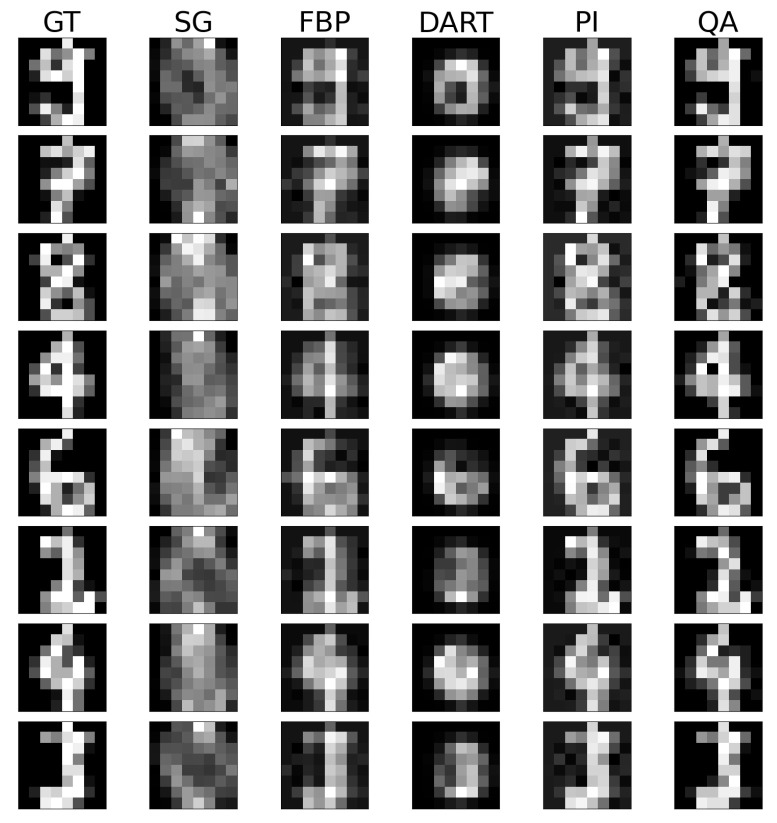
4-bit integer reconstructions of digits from the UCI digits dataset.

**Figure A7:**
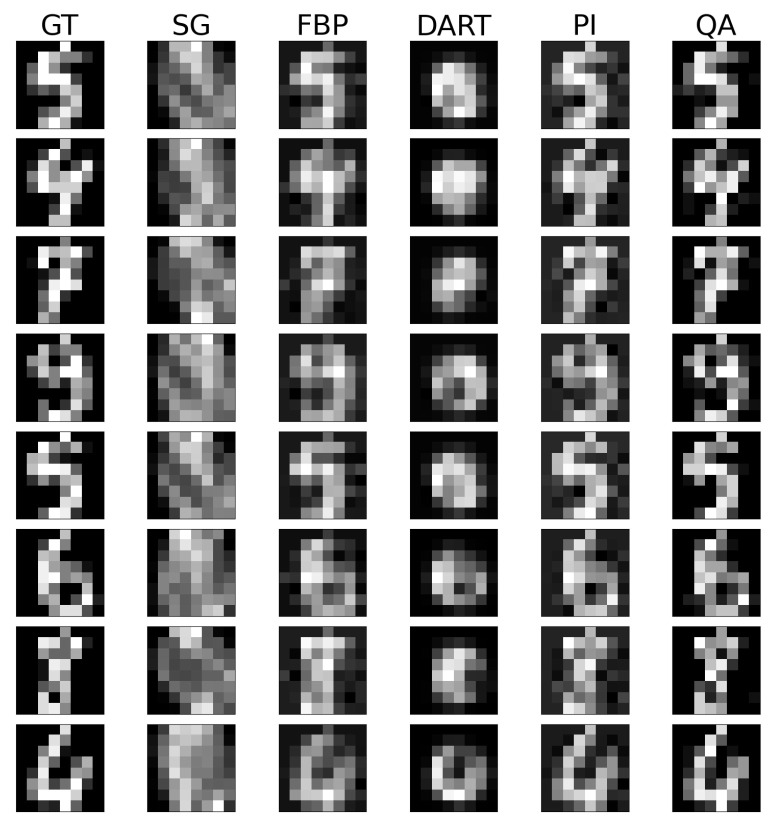
4-bit integer reconstructions of digits from the UCI digits dataset with random noise.

**Figure A8:**
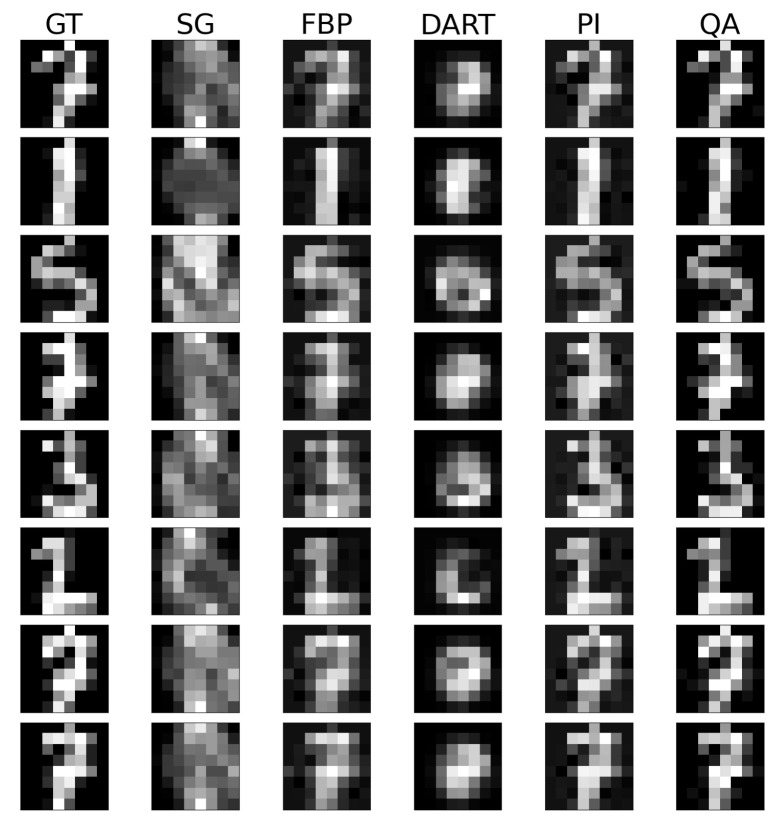
4-bit integer reconstructions of digits from the UCI digits dataset with random noise.

**Figure A9:**
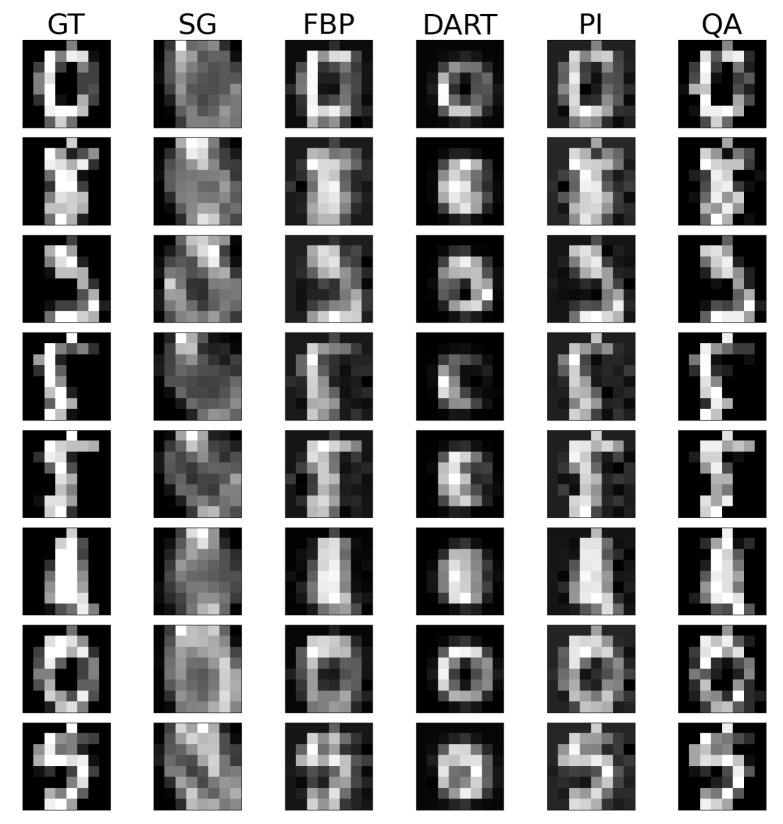
4-bit integer reconstructions of digits from the UCI digits dataset with random noise.

**Figure A10:**
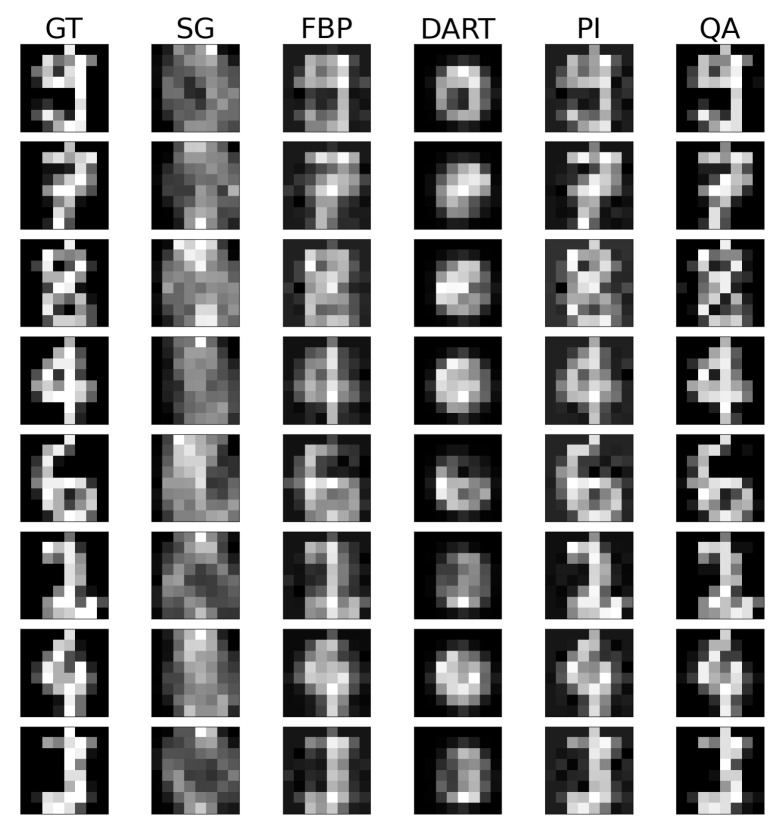
4-bit integer reconstructions of digits from the UCI digits dataset with random noise.

**Figure A11:**
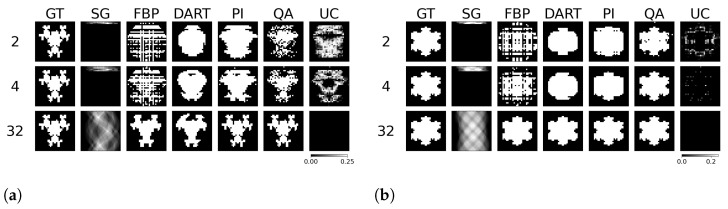
Binary reconstruction of the 32×32 image ‘molecule’ (**a**) and ‘snowflake’ (**b**) from 2, 4, and 32 views.

**Figure A12:**
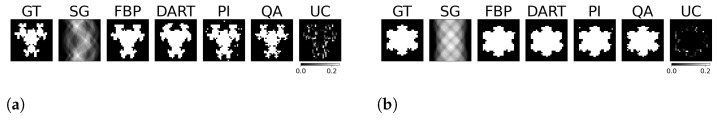
Outside of the inverse-crime scenario: Binary reconstructions of sample image ‘molecule’ (**a**) and ‘snowflake’ (**b**) for image size 32×32. The projections are simulated on an upscaled higher-resolution image 128×128 of the input, and the sinogram is consequently rebinned.

## Data Availability

The data and code used to simulate and reconstruct the tomographic data using a quantum annealer and associated hybrid solvers is available at https://github.com/merlzbert/Quantum-Annealing-Emission-Tomography (accessed on 28 August 2023). Correspondence and requests for materials should be addressed to M.A.N.

## Abbreviations

AQC	Adiabatic Quantum Computing
CQM	Constrained Quadratic Model
DART	Discrete Algebraic Reconstruction Technique
ET	Emission Tomography
FBP	Filtered BackProjection
GT	Ground Truth
HHL	Harrow-Hassidim-Lloyd
PI	Pseudo Inverse
QA	Quantum Annealing
QAOA	Quantum Approximate Optimization Algorithm
QC	Quantum Computing
QFT	Quantum Fourier Transform
QFT	Quantum Fourier Transform
QPU	Quantum Processing Unit
QUBO	Quadratic Unconstrained Binary Optimization
RMSE	Root Mean Square Error
SART	Simultaneous Algebraic Reconstruction Technique
SG	Sinogram
SSIM	Structural Similarity Index Measure
TT	Transmission Tomography
UC	Uncertainty Map
